# Corrigendum to “The correlation between sub‐epidermal moisture measurement and other early indicators of pressure ulcer development‐A prospective cohort observational study. Part 1. The correlation between sub‐epidermal moisture measurement and ultrasound”

**DOI:** 10.1111/iwj.14877

**Published:** 2024-04-15

Wilson HJE, Patton D, Budri AMV, Boland F, O'Connor T, McDonnell CO, Rai H, Moore ZEH. The correlation between sub‐epidermal moisture measurement and other early indicators of pressure ulcer development‐A prospective cohort observational study. Part 1. The correlation between sub‐epidermal moisture measurement and ultrasound. *Int Wound J*. 2024 Mar;21(3):e14732. doi: 10.1111/iwj.14732.

In the abstract, the second sentence incorrectly describes the article as a “three‐part” series. It should have been described as a “two‐part” series.

We apologize for this error.

